# Hypertension in a young boy: an energy drink effect

**DOI:** 10.1186/1756-0500-5-591

**Published:** 2012-10-29

**Authors:** Asma Usman, Ambreen Jawaid

**Affiliations:** 1 Department of Family Medicine, Aga Khan University, Karachi, Pakistan

**Keywords:** Energy drinks, Caffeine, Sinus tachycardia, High blood pressure

## Abstract

**Background:**

Use of energy drinks has significantly increased in recent times. Besides athletes, teenagers and students are among the most common consumers. However, popularity is also increasing among the younger and older age groups. Most of the users believe that they are a good source of instant energy and are unaware of its high Caffeine content resulting in harmful effects on health.

**Case presentation:**

We report the case of a young boy who presented with palpitations and high blood pressure as a result of energy drinks usage. He had been consuming a “Sting” energy drink on regular basis while studying for long hours during his O’ level Exams. His medical examination revealed Sinus tachycardia and high blood pressure. Rest of the examination and lab workup was within normal limits. His pulse and blood pressure returned to normal range after discontinuing Sting usage.

**Conclusion:**

Several studies have reported numerous health hazards including cardiac effects associated with energy drinks. Warning labeling should be done on these drinks regulating the content of Caffeine and its harmful effects on health.

## Background

Since the debut of Red Bull, the current worldwide leader of energy drinks, which was introduced in Austria in 1987 and in United States in 1997, the energy drink market has grown tremendously. Consumption of energy drinks is a common practice among college and high school students and athletes. In a survey of college students in Central Atlantic region of United States, 51% reported consuming at least one energy drink in the last month [1]. A study conducted on Pre Examination stress in second year students of Dow Medical college, from Pakistan showed that 38.94% students had increased consumption of Caffeine and energy drinks to cope up with stress [2].

People are usually not aware of the combined effects of Caffeine with other products in energy drinks, nor are aware of the amount of Caffeine in various brands. These drinks are attractive especially for our male teenage population and are easily available at local shops, big grocery stores, gas stations, clubs and even food courts of shopping malls. Different brands of energy drinks available in Pakistan include Sting, Red bull, Power Horse, Gold Street, Firer Horse etc.

Children and adolescents who are not regular Caffeine users are prone to Caffeine intoxication due to absence of pharmacological tolerance [3].

## Case presentation

We report the case of 16 years old boy, who presented to Aga Khan University hospital family physician clinic with complaints of palpitations off and on for last one week. He had also taken his blood pressure readings for the past one week. His readings were in the range of 140-160/ 80–100 mm Hg. Previously his blood pressures had always been normal. His history revealed that he had consumed about 80–100 cans of “Sting” energy drink in the past 2 weeks, an average of 3 cans per day while studying for his O’ level Exams. His rest of the history was unremarkable. Examination revealed a regular pulse of 110/min and B.P of 150/95mm Hg. There was no radio femoral delay or renal bruit. Rest of the general and systemic exam was unremarkable. His labs were ordered to rule out secondary causes of hypertension. CBC, FBS, Lipids, Cr, Na+, K+, UDR, TSH, VMA levels and ECG were all within normal range. He was advised to abstain from energy drinks use and to monitor his blood pressure. His readings returned to normal and palpitations got resolved within 2 weeks of discontinuing energy drinks’ usage.

## Discussion

“Energy drinks” are beverages that contain caffeine, taurine, ginseng, guarana, vitamins, herbal supplements, and sugar [4]. They are usually merchandised to improve energy, performance, and concentration spans. The market for energy drinks is continually growing and the annual worldwide energy drink consumption is increasing [5].

According to self report surveys, energy drinks are consumed by 30 to 50% adolescents and young adults. They are available in around 140 countries in the world including Pakistan [6]. Globally, young adults especially students and athletes are primary targets of campaigns carried out by energy drink companies. They are frequently consumed by athletes prior to competitions with a view to improve their performance [7]. They are also consumed by a large number of students especially while studying for Exams to cope with stress. In a survey of 496 college students conducted in Central Atlantic region of Unites States, 51% reported consuming at least one energy drink in the last month. It was found that 67% used it for insufficient sleep, 65% used it for insufficient energy, and 54% used it with alcohol in parties. Out of all, 22% reported having headaches after it and 19% had palpitations [1]. In our case, he was also a student who had consumed it for remaining awake whole night studying for his Exams.

Caffeine is the main active ingredient in energy drinks and its concentration is almost 3 times that in cola drinks but it can go up to 5 times, because of added products like guarana, cocoa and kola nut [8]. Guarana is a plant that contains caffeine, theobromine and theophylline which are chronotropes and ionotropes respectively [6]. Doses of caffeine found in energy drinks can range from 80 to 300 mg in a 240 ml-ounce serving. However, some brands are sold in 500 or 700 ml sizes, which increases the chances of caffeine consumption [8]. The FDA presently regulates the amount of caffeine in soft drinks to a specific maximal dosage and also requires warning instructions to be written on over the counter stimulant medications, but energy drinks are not subjected to the same regulations though they contain much higher caffeine content [3]. The product “Sting” which our case consumed is the local version and its 500 ml (2 serving) bottle does have a label of ingredients including caffeine. It also has a small precaution for children and pregnant women, but the message is not as strong as the health warning on smoking packs. However, the 250 ml (single serving) bottle does not have any ingredients label and health warning, at all.

Caffeine intoxication is a documented clinical syndrome included in the Diagnostic and Statistical Manual of Mental Disorders (DSM-IV) and the World Health Organization’s International Classification of Diseases(ICD-10) [9]. Excessive caffeine consumption has been found to have injurious health consequences. A pilot study conducted on 18–45 years old healthy, normotensive, non smoking subjects found that single day energy drink supplementation increased mean 24-hour and daytime blood pressure compared to caffeine control [10]. A similar study conducted on 15 individuals of 18–40 years age group showed that consumption of 2 cans of energy drinks daily for one week increased heart rate by 5–7 beats per minute and systolic blood pressure increased by 10 mm Hg [11]. The above 2 studies show that excessive energy drink consumption can lead to hypertension as seen in our case.

Cardiac arrest was reported in a 28 year old athelete who had consumed excessive amount of energy drinks containing caffeine [12]. 3 cases of new onset seizures were reported in adults associated with tachycardia and systolic hypertension which did not re occur after the patients remained abstinent from energy drinks for a few months [13]. Even death has also been reported in four documented cases due to excessive energy drinks consumption [8]. A double blind study conducted on 13 endurance trained participants showed that original RED BULL drink containing taurine increases cardiac contractility [14]. Since caffeine is also a diuretic and these energy drinks are usually consumed by children and adolescence before sports event, the combined effect of sweating and diuresis can lead to severe dehydration [15]. In Pakistan also, numerous children and adolescent athletes consume them before and after sport activities to boost their energy. Majority of them are totally unaware of their ingredients and their harmful effects as there is no clear labeling of health warning over them. Our case was also not aware that “Sting” had caffeine and about its harmful health effects.

Producers of energy drinks usually target young adults who are easily lured to consume energy drinks after watching numerous appealing marketing advertisements on television, in newspapers and magazines [16]. Regulation of energy drinks, including content labeling and health warnings has differed across countries. USA has the most lax regulatory requirements, and is also the largest market for these products. The absence of regulations has resulted in aggressive marketing of energy drinks [3]. And there is absolutely no check in Pakistan on import, sale and content regulation of these energy drinks.

## Conclusion

There are numerous false perceptions in the society about the positive benefits and harmful effects of energy drinks. There is a strong need to create awareness through health education regarding these drinks especially among children as they are exposed to an ever-increasing range and easily accessible energy drinks market. There is also a strong need of legislation regarding mandatory labeling of exact caffeine content of these drinks and with strong health warning regarding potential health risks. These health warnings must also be included in TV commercials and print media advertisements.

## Consent

"Written informed consent was obtained from the patient’s mother, as patient is a minor, for publication of this Case report. A copy of the written consent is available for review by the Editor of this journal."

## Abbreviations

B.P: Blood Pressure; CBC: Complete Blood Count; FBS: Fasting Blood Sugar; Cr: Creatinine; Na+: Sodium; K+: Potassium; UDR: Urine Detailed Report; TSH: Thyroid Stimulating Hormone; VMA: Venyl Mandelic Acid; ECG: Electrocardiogram; FDA: Food and Drug Administration.

## Competing interests

The authors declare that they have no competing interests.

## Authors’ contributions

AU managed the reported patient, took consent and conceived the idea of writing it. Both AU an AJ did the literature search and drafted the manuscript. AU gave the final approval of the version to be published. Both authors read and approved the final manuscript.

## Authors’ information

Asma Usman is working as a lecturer in the Department of Family Medicine, Aga Khan University. (asma.usman@aku.edu)

Ambreen Jawaid is working as a Resident in the Department of Family Medicine, Aga Khan University. (ambreen.jawaid@aku.edu)
